# Assessment of bioaerosol composition and public health implications in high-traffic urban areas of Southwest, Nigeria

**DOI:** 10.1088/2515-7620/ad9e87

**Published:** 2024-12-24

**Authors:** Daniel Abayomi Odeyemi, Jude Oluwapelumi Alao, Tolulope Adeyemi Kayode, Ernest Uzodimma Durugbo

**Affiliations:** 1 The School of Medicine, Dentistry and Biomedical Sciences, Faculty of Medicine, Health and Life Sciences, Queen’s University Belfast, United Kingdom; 2 African Centre of Excellence for Genomics of Infectious Diseases (ACEGID), Redeemer’s University, Ede, Osun State, Nigeria; 3 School of Public Health and Interdisciplinary Studies, Auckland University of Technology, Auckland, New Zealand; 4 Department of Biological Sciences, University of Notre Dame, Holy Cross Dr, Notre Dame, IN, United States of America; 5 Department of Biological Sciences, College of Natural Sciences, Redeemer’s University, Ede, Osun State, Nigeria

**Keywords:** bioaerosols, public health, air quality, pathogenic microorganisms, Nigeria

## Abstract

Bioaerosols, a significant yet underexplored component of atmospheric particulate matter, pose substantial public health risks, particularly in regions with poor air quality. This study investigates the composition of bioaerosols in public spaces, specifically two interstate motor parks and two marketplaces in Osun State, Nigeria, over six months. Air samples were collected, and bacterial and fungal species were identified, focusing on pathogenic organisms. The results revealed the presence of well-known pathogens, including *Staphylococcus aureus, Escherichia coli, Klebsiella sp., Pseudomonas aeruginosa, Aspergillus sp*., and *Fusarium sp*., which are associated with respiratory and gastrointestinal infections, as well as antimicrobial resistance. Site-specific differences in microbial diversity were observed, with higher bacterial diversity in motor parks and greater fungal occurrence in marketplaces influenced by environmental factors such as waste management. The findings highlight the urgent need for microbial air quality monitoring in public spaces, alongside improved sanitation practices. This study provides critical insights into the public health risks posed by bioaerosols and calls for local and global interventions to mitigate the impact of airborne pathogens in urban environments.

## 1. Introduction

Bioaerosols, a subset of atmospheric particulate matter, are composed of biological materials such as bacteria, fungi, viruses, pollen, and living organism fragments [1–3]. Originating from both terrestrial and marine ecosystems, bioaerosols are ubiquitous in the atmosphere and play an important yet underexplored role in environmental and public health. The transport and dispersion of bioaerosols through natural processes like wind and precipitation can carry pathogens and allergens over long distances, raising concerns about their potential health impacts [1], particularly in regions already burdened by poor air quality.

Once airborne, bioaerosols interact dynamically with other particulate matter and water droplets in clouds and precipitation, allowing them to traverse vast areas before being redeposited through dry and wet deposition processes [4, 5]. Their ability to travel significant distances amplifies their reach, increasing human exposure to pathogenic species and allergens that may contribute to respiratory diseases, including asthma and chronic obstructive pulmonary disease (COPD) [6, 7]. These concerns are particularly relevant in the context of global air pollution, which is responsible for 4.2 million premature deaths annually [8], with a considerable portion linked to respiratory conditions exacerbated by airborne pollutants, including bioaerosols.

Recent studies have heightened awareness of bioaerosols’ role in public health, especially their contribution to the burden of respiratory diseases. Bioaerosols can carry microorganisms and allergens capable of triggering adverse health effects upon inhalation. Among the most vulnerable are individuals in regions with pervasive air pollution, such as sub-Saharan Africa. Here, outdoor air pollution is a significant public health issue driven by rapid urbanisation, industrial emissions, and poor waste management practices [9]. The region’s high incidence of Acute Lower Respiratory Infections (ALRIs) underscores the urgency of understanding bioaerosol composition in relation to air quality and public health outcomes [10].

In Nigeria, urban centres face mounting air quality challenges due to increasing industrialisation, vehicular emissions, and inadequate pollution control measures. Studies from Nigerian cities, including Lagos and Zaria, have documented particulate matter concentrations far exceeding World Health Organization (WHO) safety thresholds [11–14]. This raises concerns about the additional presence of bioaerosols, especially given the documented rise in respiratory illnesses in African countries [9, 15]. Despite the growing body of research on chemical air pollutants, microbial air pollutants such as bioaerosols remain underexplored in Nigeria, creating a knowledge gap in the relationship between airborne microorganisms and public health.

This six-month study will investigate bioaerosol diversity in Osun State, Southwestern Nigeria, focusing on two interstate motor parks and two large marketplaces in Ede and Osogbo. These locations are selected for their high foot and vehicular traffic, and environmental conditions that may contribute to varied microbial activity. Through the systematic collection, isolation, and identification of bacteria and fungi, with a focus on potential pathogenic species, the study is expected to provide valuable insights into the microbial composition of urban air. In addition to identifying potential microbial hazards, the study aims to offer recommendations for improving air quality monitoring, enhancing sanitation in high-traffic areas, and strengthening public health policies to mitigate bioaerosol-related risks in urban environments.

## 2. Materials and methods

### 2.1. Sample collection locations

Microbial samples were collected from the ambient air at two specific motor parks in Osun State, Nigeria:

• Ola-Iya Junction, Osogbo, Osun State, Nigeria Coordinates: 7°46'33.0 ‘N 4°32'44.1 ‘E
• Akoda Junction, Ede, Osun State, Nigeria Coordinates: 7°40'41.1 ‘N 4°27'48.2 ‘E

These locations were selected due to the high intensity of human and vehicular traffic, a known factor influencing bioaerosol levels. Both sites also have various roadside food sellers, which may further contribute to the microbial diversity in the air. Akoda Junction is situated close to farmlands, while Ola-Iya Junction is located in a more metropolitan area, providing a contrast in environmental conditions. While neither site is close to major waste disposal areas, frequent animal droppings and human littering at both locations could influence microbial activity. This selection aims to reflect real-world conditions in busy urban environments, offering a nuanced understanding of how factors such as traffic, food vending, and local environmental characteristics can impact microbial profiles in high-traffic areas.

### 2.2. Sample collection phase

Standard Petri dishes containing the culture media were exposed to the air for 10 to 15 min [16] once every two weeks, around 2 p.m., between October 2019 and March 2020, totalling 12 weeks of sampling. After exposure, the plates were sealed and transported to the microbiology laboratory at Redeemer’s University, Nigeria, for further analysis. The sealed plates were incubated at 37 °C for 24 h to isolate bacterial species and at 25 °C for 48 h to isolate fungal species.

The sampling conditions, including the average temperature and humidity during each week of collection, are as follows:

• **October**: Week 1–28 °C, 100% humidity; Week 2–24.5 °C, 97% humidity
• **November**: Week 3–28 °C, 92% humidity; Week 4–29 °C, 74% humidity
• **December**: Week 5–29 °C, 60% humidity; Week 6–30 °C, 41% humidity
• **January**: Week 7–28.5 °C, 38% humidity; Week 8–30 °C, 47% humidity
• **February**: Week 9–29.5 °C, 58% humidity; Week 10–31 °C, 69% humidity
• **March**: Week 11–30.5 °C, 82% humidity; Week 12–31 °C, 90% humidity

Information on the bacterial isolates is provided in Supplementary File 1, while details on the fungal isolates can be found in Supplementary File 2.

### 2.3. Rationale for selection of pathogenic species

The selection of specific pathogenic species for microbial analysis was based on their known health risks and prevalence in urban environments [17]. These pathogens are associated with respiratory infections and other health issues, making them significant for assessing public health risks related to bioaerosols in Osun State, Nigeria.

### 2.4. Preliminary identification tests for bacterial isolates

After isolating pure bacterial cultures, a series of preliminary identification tests were performed to characterise the bacterial species:

1. *Gram staining:* To determine the Gram reaction and morphology of the bacterial cells, the bacterial cells were fixed to a slide, stained with crystal violet (Sigma-Aldrich, USA), treated with iodine solution (Sigma-Aldrich, USA), decolourised (95% Ethanol), and counterstained with safranin (Sigma-Aldrich, USA). The stained cells were examined under a light microscope to identify Gram-positive (purple) and Gram-negative (pink) bacteria.
2. *Motility Test:* To determine whether the bacteria are motile or non-motile, bacterial culture was inoculated into the centre of the motility medium (Oxoid, UK). After incubation, motility was indicated by the diffuse growth spreading from the stab line.
3. *Biochemical tests:* The following biochemical tests were done to identify bacterial species based on metabolic and enzymatic activities:
  • *Catalase Test:* 3% Hydrogen Peroxide (Sigma-Aldrich, USA) was added to the bacterial culture. Bubble formation indicated a positive catalase reaction.
  • *Indole Test:* Kovac’s Reagent (Oxoid, UK) was added to the culture after incubation. A red colour at the top of the broth indicated a positive indole test.
  • *Citrate Utilization Test:* Bacteria were inoculated into Simmons Citrate Agar (Oxoid, UK), and the colour change from green to blue indicated a positive citrate utilisation test.
  • *Sugar Fermentation Tests:* The bacteria were inoculated into Glucose, Sucrose, Lactose, and Mannitol Broths (Oxoid, UK) containing different sugars and phenol red as an indicator—the colour change to yellow indicated sugar fermentation.

4. *MR-VP test:* Methyl Red Reagent (Sigma-Aldrich, USA) was added to test for acid production (red for positive M.R. test), and Voges-Proskauer Reagents (Oxoid, UK) were added to detect acetoin (red colour after 30 min for positive V.P. test).
5. *Starch hydrolysis test:* An iodine solution was added to Starch Agar (Oxoid, UK) after incubation. A clear zone around the bacterial growth indicated starch hydrolysis.

### 2.5. Molecular techniques for bacterial identification

To further identify bacterial isolates, several molecular techniques were employed:

1. *DNA extraction:* DNA was extracted from bacterial cultures using the DNA Extraction Kit (Qiagen, Germany). Bacterial cells were lysed, and the DNA was bound to a column. After removing impurities, the DNA was eluted and collected for polymerase chain reaction (PCR).
2. *PCR amplification: method:* The 16S rDNA gene was amplified using the 16S rDNA Gene Amplification Kit (Promega, USA).
3. *Primers used:*
  27F: 5’-AGAGTTTGATCCTGGCTCAG-3’
  1492R: 5’-GGTTACCTTGTTACGACTT-3’ [18]
  PCR reactions were set up with the extracted DNA, primers, and a PCR master mix. The reaction conditions included an initial denaturation step, followed by multiple cycles of denaturation, annealing, and extension to amplify the 16S rDNA gene.
4. *Agarose gel electrophoresis:* The PCR products were analysed using 2% agarose gel electrophoresis prepared with TAE Buffer (Bio-Rad), USA, and visualised using GelRed Nucleic Acid Stain (Biotium, USA). The gel was prepared by dissolving agarose in TAE buffer and adding GelRed for DNA staining. The PCR products were loaded into the gel wells, and an electric current was applied to separate the DNA fragments by size. The gel was then viewed under U.V. light to confirm the successful amplification of the 16S rDNA gene.
5. *Sanger sequencing:* The amplified 16S rDNA gene products were sent to the African Centre of Excellence for Genomics of Infectious Diseases (ACEGID) in Nigeria for Sanger Sequencing. The sequencing process determined the nucleotide sequence of the PCR products, providing the genetic information necessary for bacterial identification.
6. *Bioinformatics analysis:* The sequencing data were analysed using the Basic Local Alignment Search Tool (BLAST) on the National Centre for Biotechnology Information (NCBI) database. The BLAST search compared the obtained 16S rDNA sequences against the NCBI database to find homologous sequences and identify the bacterial species based on sequence similarity.

### 2.6. Phylogenetic analysis of 16S rDNA genetic sequences

The phylogenetic analysis of the 16S rDNA genetic sequences was performed to determine the evolutionary relationships of the bacterial isolates. The methodology involved several key steps:

1. *BLAST search:* The 16S rDNA gene sequences of the bacterial isolates were compared against the NCBI database using BLAST. The BLAST search was configured to identify sequences with the highest percentage similarity, targeting an E-value of 0.0 and a query coverage greater than 96% to ensure high-confidence matches.
2. *Data retrieval and sequence alignment:* The BLAST results were downloaded in FASTA format. Sequences with the highest similarity to the query sequences were selected for further analysis.
3. *Phylogenetic tree construction:* The Maximum Likelihood method was employed for tree construction, utilising the Tamura-Nei model for evolutionary distance estimation [19]. This model was chosen for its robustness in accounting for nucleotide substitutions. The Neighbor-Join (N.J.) and BioNJ algorithms generated initial trees based on pairwise distances calculated using the Tamura-Nei model [19]. The topology with the highest log-likelihood value was selected as the best-fit model. All codon positions (1st, 2nd, 3rd, and noncoding) were included in the analysis [20]. A Bootstrap test with 1000 replicates was conducted to assess the reliability of the phylogenetic tree [21]. The Bootstrap values were used to evaluate the robustness of the tree branches. The final phylogenetic tree was visualised and annotated using Molecular Evolutionary Genetics Analysis Version X (MEGA X). The tree illustrates the evolutionary relationships between bacterial isolates and their closest relatives.

### 2.7. Identification of fungal isolates

The identification of fungal isolates was based on a combination of morphological and cultural features observed under a light microscope (Olympus Corporation, Japan). Fungal material was mounted on a slide with glycerine-lactophenol (Sigma-Aldrich, USA) and examined at x10 and x40 magnifications. The fungi isolates were grouped into A-E based on their phenotypic characteristics, such as spore shape, spore colour, surface texture, and growth pattern were documented and used for identification. Table 1 summarises the phenotypic characteristics of each fungal isolate and the corresponding identification references.

**Table 1.** Phenotypic characteristics and identification of fungal isolates.

Fungal isolate	Spore shape	Spore colour	Surface texture	Growth pattern	Identification references
Isolate A	Round	Green	Smooth	Radial	[22]
Isolate B	Ellipsoidal	White	Rough	Uniform	[23]
Isolate C	Cylindrical	Black	Velvety	Circular	[24]
Isolate D	Oval	Yellow	Powdery	Spreading	[25]
Isolate E	Spherical	Pink	Fuzzy	Irregular	[25]

## 3. Results

### 3.1. Identification of bacteria isolated from different locations

All bacterial isolates that grew on nutrient agar media were sub-cultured to obtain pure cultures for storage and identification. The total bacterial occurrence per site, isolated from motor parks and marketplaces, is presented in table 2. The term ‘total occurrence’ refers to the number of bacterial colonies observed at each site, representing the cumulative count of bacterial isolates. Additionally, table 3 provides useful information, showing the total microbial count (cfu/ml) of growth observed per week at each location, illustrating the variability in microbial presence across the 12-week period at Ola-Iya Junction (OLA) and Akoda Park (AKD). This count was used to further assess bacterial concentration trends over time.

**Table 2.** Table displaying the total microbial count (cfu/ml) recorded each week, along with the corresponding locations where the organisms were isolated.

Location	Week 1	Week 2	Week 3	Week 4	Week 5	Week 6	Week 7	Week 8	Week 9	Week 10	Week 11	Week 12
OLA	79	84	35	50	38	18	31	39	21	45	41	51
AKD	93	143	245	180	248	175	203	183	203	235	189	191

**Table 3.** Assessment of bacterial occurrence at interstate marketplaces and travel motor parks.

Microorganisms	Travel motor parks		Interstate marketplaces	
	AKD	OLA	AKD	OLA
*Bacillus cereus*	3	3	5	5
*Bacillus altitudinis*	1	1	—	—
*Escherichia coli*	4	4	3	3
*Staphylococcus aureus*	5	0	3	3
*Streptomyces sp.*	0	3	5	5
*Pseudomonas aeruginosa*	3	1	1	1
*Klebsiella sp.*	2	2	1	1
*Bacillus pumilus*	2	1	1	1
*Rhodococcus pyridinivorans*	2	2	1	1
Total	22	17	20	20

*AKD: Akoda, OLA: Olaiya.

### 3.2. Result of PCR amplification of bacterial 16S rDNA

**Figure 1.**
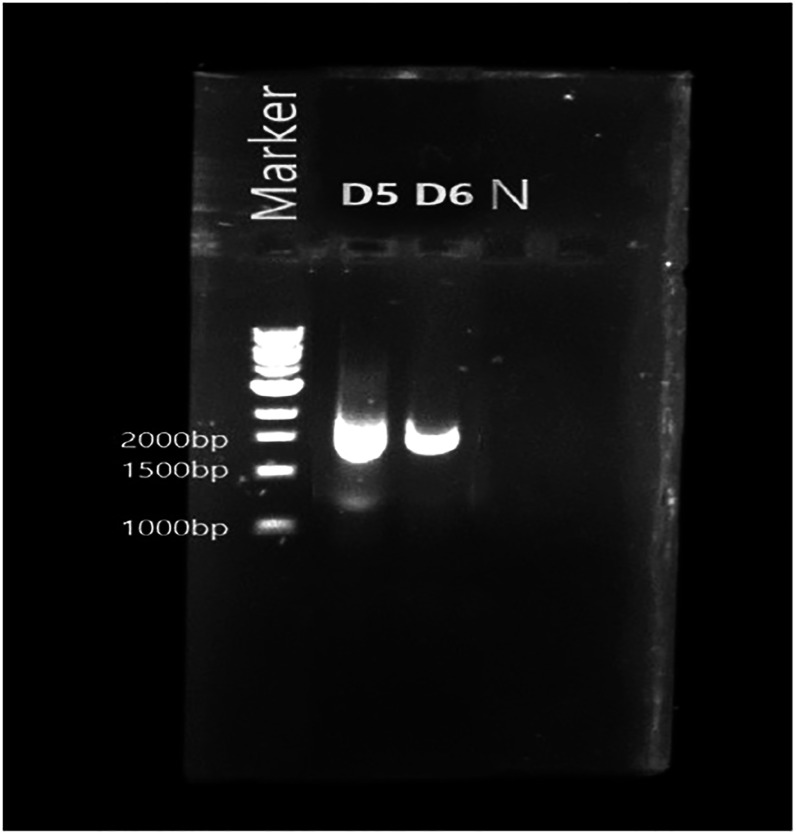
An agarose gel image representing the PCR amplicons of the 16S rDNA. The gel was prepared using a 2% agarose concentration and visualised the amplified DNA fragments resulting from the PCR reaction.

Figure 1 depicts the results of a 2% agarose gel electrophoresis, displaying the PCR amplicons obtained from this procedure.

### 3.3. Results of bacterial 16 s rDNA sequencing

The details of the sequence identification, top BLAST hits, percentage identity, and query coverage are provided in table 4.

**Table 4.** The sequence analysis results include the sequence identity, top BLAST hits, percentage identity, and query coverage against Genbank (NCBI).

Sequence ID	Top blast hit (s)	Percentage identity (%)	Query coverage	Source
D5	*Rhodococcus pyridinivorans*	96.07	97	OLA
D6	*Bacillus pumilus*	99.59	98	AKD

### 3.4. Results of phylogenetic analysis of 16 s rDNA genetic sequences

**Figure 2.**
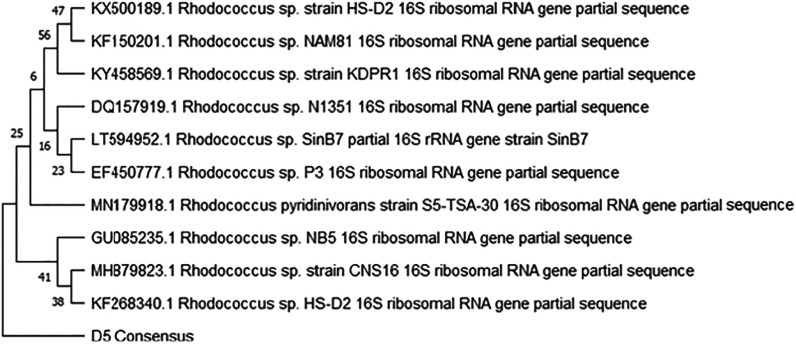
The phylogenetic tree shows the relationship between isolated D5 labelled ‘D5 Consensus Sequence’ (*Rhodococcus pyridinivorans*) and highly identical reference genomes from the NCBI.

**Figure 3.**
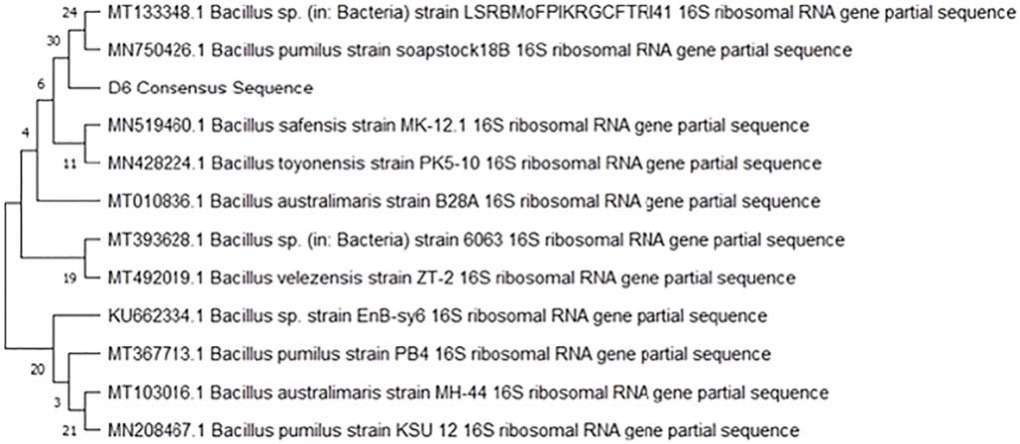
The phylogenetic tree shows the relationship between isolated D6 labelled ‘D6 Consensus Sequence’ (*Bacillus pumilus*) and highly identical reference genomes from the GenBank (NCBI).

Figures 2 and 3 display the phylogenetic trees illustrating the relationship between each isolate and highly identical reference genomes obtained from NCBI. The analysis revealed that sample D5 corresponds to *Rhodococcus pyridinivorans*, while sample D6 corresponds to *Bacillus pumilus* (Supplementary File 3). The identification was based on comparing genetic data from the sequencing with known sequences in the database.

*The evolutionary history was inferred using the Maximum Likelihood method and the Tamura-Nei model. The tree with the highest log likelihood (*−*2340.37) is shown. The percentage of trees in which the associated taxa clustered is shown next to the branches. Initial tree(s) for the heuristic search were obtained automatically by applying Neighbour-Join and BioNJ algorithms to a matrix of estimated pairwise distances using the Tamura-Nei model, and then the topology with superior log likelihood value was selected. This analysis involved 11 nucleotide sequences. Codon positions included were 1st+2nd+3rd+Noncoding. There were a total of 1533 positions in the final dataset.*

*The evolutionary history was inferred by using the Maximum Likelihood method and the Tamura-Nei model. The tree with the highest log likelihood (*−*2394.69) is shown. The percentage of trees in which the associated taxa clustered together is shown next to the branches. Initial tree(s) for the heuristic search were obtained automatically by applying Neighbour-Join and BioNJ algorithms to a matrix of estimated pairwise distances using the Tamura-Nei model, and then the topology with superior log likelihood value was selected. This analysis involved 12 nucleotide sequences. Codon positions included were 1st+2nd+3rd+Noncoding. There was a total of 1*5*24 positions in the final dataset.*

### 3.5. Identification of fungi isolated from different locations

**Figure 4.**
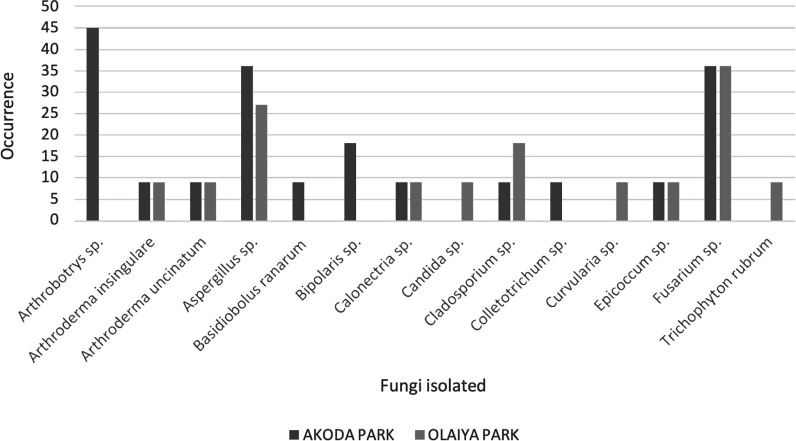
Occurrence of isolated fungi per location sampled.

The identified isolated from the sampled locations during this study include *Candida sp., Aspergillus sp., Fusarium sp., Epicoccum spp., Cladosporium spp., Trichophyton rubrum, Arthroderma uncinatum, Curvularia spp., Fusarium spp*., and *Cladosporium spp.* Specifically, *Arthrobotrys spp*. and *Basidiobolus ranarum* were isolated exclusively from Akoda Park but not found in Olaiya Park. Conversely, *Trichophyton rubrum, Curvularia sp.*, and Candida sp. were isolated from Olaiya Park but absent in Akoda Park. A comparison of the study isolates is shown in figure 4.

## 4. Discussion

According to the WHO, 99% of the world’s population lives in areas where air quality fails to meet recommended standards. Ambient air pollution is responsible for 6.7 million premature deaths annually, with respiratory illnesses being a significant contributor to this figure [8]. Bacteria, a critical component of bioaerosols, have been widely studied for their role in air pollution. This study provides an in-depth examination of bioaerosol composition in Osun State, Nigerian public spaces, revealing both pathogenic and environmental bacterial and fungal species that pose significant public health risks.

This study’s bacteria isolated from motor parks and marketplaces include several well-known human pathogens, such as *S. aureus, E. coli, Klebsiella sp.,* and *P. aeruginosa*. These bacteria are of particular concern due to their association with severe infections, including respiratory, gastrointestinal, and skin diseases, and their involvement in antimicrobial resistance (AMR) [26]. The prevalence of *S. aureus* and *E. coli* across both motor parks and marketplaces aligns with findings from similar studies in urban environments, where these pathogens are frequently detected in bioaerosols. For instance, studies in urban areas of Asia and Europe have also reported high levels of *S. aureus* in crowded public spaces, emphasising the role of bioaerosols in pathogen dissemination [27–29]. Additionally, *E. coli* has been frequently identified in bioaerosol studies conducted in densely populated urban settings [30, 31], highlighting the global concern of bacterial contamination in such environments. By drawing comparisons with global trends, this study underscores the significance of public spaces, such as motor parks and marketplaces, as reservoirs for airborne pathogens, increasing the risk of transmission in crowded, high-traffic environments.

The isolation of *Klebsiella sp*. and *P. aeruginosa* further emphasises the potential risk of AMR spreading via bioaerosols. *Klebsiella sp*., often implicated in hospital-acquired infections, is crucial in pneumonia and bloodstream infections [32], while *P. aeruginosa* is notorious for its role in the infection of immunocompromised individuals [33]. Given the increasing burden of AMR in Nigeria, these resistant organisms in ambient air raise concerns about the spread of multidrug-resistant strains in community settings.

The phylogenetic analysis of the bacterial species, including identifying *R. pyridinivorans* and *B. pumilus*, indicates that some bacteria in this study could have dual roles as pathogens and environmental organisms. For example, *B. pumilus* has been linked to foodborne illness outbreaks but is also utilised in agricultural applications due to its fungicidal properties [34, 35]. This dual nature underscores the complexity of bioaerosols and the necessity for comprehensive monitoring of both pathogenic and beneficial microorganisms in the air.

The fungal isolates identified, including *Aspergillus spp., Fusarium sp.,* and *Candida sp.,* also present serious health concerns, particularly for individuals with pre-existing respiratory conditions such as asthma or COPD. *Aspergillus sp.,* which was detected in both motor parks and marketplaces, is a common cause of allergic bronchopulmonary aspergillosis and invasive aspergillosis in immunocompromised populations [36]. The isolation of *Fusarium spp.* highlights another emerging threat, as this fungus is increasingly recognised as an opportunistic pathogen, especially in hospital settings where it can cause infections in patients with weakened immune systems [37].

The site-specific differences in fungal composition, with species like *Arthrobotrys sp*. and *B. ranarum* isolated exclusively from Akoda Park, suggest that environmental factors, such as proximity to agricultural activities and waste disposal practices, play a significant role in shaping bioaerosol composition. This variability indicates that localised interventions, such as improving waste management and controlling organic material accumulation, could reduce fungal spore concentrations and minimise respiratory infection risk.

The higher microbial diversity observed in motor parks compared to marketplaces could be attributed to several factors, including vehicular emissions, human density, and environmental conditions such as temperature and humidity. Motor parks, which experience heavy foot traffic and vehicular movement, provide an ideal setting for the dispersion of bioaerosols. The presence of *S. aureus* and *E. coli* in these locations, both commonly associated with human activity, underscores the role of human interaction in shaping microbial air content.

In contrast, the higher occurrence of fungal species in marketplaces likely reflects the influence of organic material and waste products. Poor waste management, coupled with high levels of food handling, creates an environment conducive to the growth and dispersion of fungal spores. The isolation of *Candida sp., Aspergillus sp*., and *Fusarium sp*. from these locations suggests that public markets may serve as significant sources of airborne fungal pathogens, particularly in regions with poor sanitation infrastructure.

Identifying pathogenic bacteria and fungi in urban spaces has significant public health implications. First, there is an urgent need to integrate microbial assessments into existing air quality monitoring programs. Current air quality standards predominantly target chemical pollutants like PM_2.5_, PM_10_, ozone, and nitrogen dioxide [38]. However, this study did not assess the influence of meteorological factors—such as wind speed, wind direction, temperature, and relative humidity—on fungal and bacterial communities. Given that Nigeria’s climate is divided into dry and wet seasons, these weather conditions likely play a significant role in bioaerosol dynamics. During the dry season, stable atmospheric conditions and reduced precipitation can lead to the accumulation of bioaerosols, while increased wind speeds during the rainy season may disperse microbial particles. Moreover, the significance of long-range transport (LRT) in bioaerosols’ occurrence, transformation, and bioavailability cannot be overlooked, as air masses are expected to carry different microbial populations [4, 39]. Analysing the origins of these air masses through 48− to 72-h back-trajectory analysis can provide valuable insights. Given the role of bioaerosols in the transmission of infectious diseases, particularly in densely populated urban areas, microbial air quality must be regularly assessed. Incorporating meteorological data and LRT analysis into these assessments would provide a more comprehensive understanding of how both chemical and biological pollutants impact public health in urban environments.

Second, the study highlights the importance of improved sanitation measures in motor parks and marketplaces. Routine cleaning, waste management, and better ventilation systems could significantly reduce the microbial load in these spaces. Educational campaigns that promote hygiene practices in public areas, particularly regarding food handling in marketplaces, should be prioritised to minimise the risk of bioaerosol-related infections.

Bioaerosols remain an underappreciated component of air pollution despite their significant contribution to disease transmission. The findings from this study emphasise the need for a concerted effort to monitor and mitigate the risks posed by bioaerosols in urban environments. As cities continue to grow and human activity increases, the risk of exposure to airborne pathogens will likely rise, particularly in regions with limited infrastructure for air quality control.

To reduce the global burden of respiratory diseases linked to air pollution, policymakers should prioritise the inclusion of bioaerosols in air quality monitoring frameworks, particularly in low- and middle-income countries where the health impacts of air pollution are most pronounced. This study demonstrates the critical need for local interventions, such as enhanced waste management and sanitation in public spaces, but it also calls for broader global action to address the threats posed by microbial air pollution.

The methodology employed for bioaerosol sampling in this study utilised standard Petri dishes; however, it is essential to note that the size distribution of the collected samples, such as PM_10_ and PM_2.5_, was not considered during sampling. This oversight may impact the compositional complexity of the biological particles detected, as different size fractions of bioaerosols can vary significantly in their origins, viability, and potential health effects. Typically, bioaerosol sampling is performed using filters with defined pore sizes, which facilitate the segregation and characterisation of particles based on their aerodynamic properties.

While modifications to the sampling procedure are not feasible at this stage, it is essential to acknowledge this limitation in our findings. The lack of size-based segregation may restrict our ability to fully understand bioaerosols’ dynamics and health implications in the studied urban environment. Future research should consider implementing more refined sampling techniques to analyse bioaerosols across various size distributions, providing a more comprehensive view of their ecological and public health significance.

## 5. Conclusion

This study underscores the public health risks posed by bioaerosols in high-traffic urban areas, with pathogenic bacteria and fungi detected in motor parks and marketplaces in Osun State, Nigeria. The findings highlight the necessity for microbial air quality monitoring, improved sanitation practices, and public health interventions to mitigate the risks associated with airborne microorganisms. Addressing bioaerosol pollution at both the local and global levels can reduce the transmission of infectious diseases and improve overall respiratory health outcomes, particularly in vulnerable urban populations.

## Data Availability

The data cannot be made publicly available upon publication because they are not available in a format that is sufficiently accessible or reusable by other researchers. The data that support the findings of this study are available upon reasonable request from the authors.
